# Novel Lytic Enzyme of Prophage Origin from *Clostridium botulinum* E3 Strain Alaska E43 with Bactericidal Activity against Clostridial Cells

**DOI:** 10.3390/ijms22179536

**Published:** 2021-09-02

**Authors:** Agnieszka Morzywolek, Magdalena Plotka, Anna-Karina Kaczorowska, Monika Szadkowska, Lukasz P. Kozlowski, Dariusz Wyrzykowski, Joanna Makowska, Jerel J. Waters, Steven M. Swift, David M. Donovan, Tadeusz Kaczorowski

**Affiliations:** 1 Laboratory of Extremophiles Biology, Department of Microbiology, Faculty of Biology, University of Gdansk, 80-822 Gdansk, Poland; a.morzywolek@gmail.com (A.M.); monika.szadkowska@phdstud.ug.edu.pl (M.S.); 2 Collection of Plasmids and Microorganisms, Faculty of Biology, University of Gdansk, 80-308 Gdansk, Poland; anna.kaczorowska@ug.edu.pl; 3 Institute of Informatics, Faculty of Mathematics, Informatics and Mechanics, University of Warsaw, 02-097 Warsaw, Poland; lukaskoz@mimuw.edu.pl; 4 Department of General and Inorganic Chemistry, Faculty of Chemistry, University of Gdansk, 80-308 Gdansk, Poland; dariusz.wyrzykowski@ug.edu.pl (D.W.); joanna.makowska@ug.edu.pl (J.M.); 5 Animal Biosciences and Biotechnology Laboratory, ARS, NEA, USDA, Beltsville, MD 20705-2350, USA; renovatebio.jerelwaters@gmail.com (J.J.W.); sswift@contrafect.com (S.M.S.); ddonovan0@yahoo.com (D.M.D.)

**Keywords:** *N*-acetylmuramoyl-l-alanine amidase, *Clostridium botulinum*, endolysin, prophage, lipoteichoic acid

## Abstract

*Clostridium botulinum* is a Gram-positive, anaerobic, spore-forming bacterium capable of producing botulinum toxin and responsible for botulism of humans and animals. Phage-encoded enzymes called endolysins, which can lyse bacteria when exposed externally, have potential as agents to combat bacteria of the genus *Clostridium*. Bioinformatics analysis revealed in the genomes of several *Clostridium* species genes encoding putative *N*-acetylmuramoyl-l-alanine amidases with anti-clostridial potential. One such enzyme, designated as LysB (224-aa), from the prophage of *C. botulinum* E3 strain Alaska E43 was chosen for further analysis. The recombinant 27,726 Da protein was expressed and purified from *E. coli* Tuner(DE3) with a yield of 37.5 mg per 1 L of cell culture. Size-exclusion chromatography and analytical ultracentrifugation experiments showed that the protein is dimeric in solution. Bioinformatics analysis and results of site-directed mutagenesis studies imply that five residues, namely H25, Y54, H126, S132, and C134, form the catalytic center of the enzyme. Twelve other residues, namely M13, H43, N47, G48, W49, A50, L73, A75, H76, Q78, N81, and Y182, were predicted to be involved in anchoring the protein to the lipoteichoic acid, a significant component of the Gram-positive bacterial cell wall. The LysB enzyme demonstrated lytic activity against bacteria belonging to the genera *Clostridium*, *Bacillus, Staphylococcus,* and *Deinococcus*, but did not lyse Gram-negative bacteria. Optimal lytic activity of LysB occurred between pH 4.0 and 7.5 in the absence of NaCl. This work presents the first characterization of an endolysin derived from a *C. botulinum* Group II prophage, which can potentially be used to control this important pathogen.

## 1. Introduction

*Clostridium botulinum* is an anaerobic, Gram-positive, spore-forming bacterium found in soil and water that causes botulism, a severe neuroparalytic disease affecting humans and animals [1]. Botulism typically results from ingestion of food containing botulinum neurotoxin (BoNT) secreted by vegetative clostridia. *C. botulinum* produces seven serotypes of botulinal neurotoxins, types A–G, divided into more than 40 different subtypes, which are the most lethal toxins known today (as little as 30 ng is sufficient to cause illness in adult humans by the oral route) [2]. Human botulism is caused mainly by toxin types A, B, E, and rarely F, while types C and D are associated with animal botulism [3]. Type A of BoNT causes the most prolonged and most severe forms of botulism, while BoNT type E leads to shorter duration symptoms, and BoNT type B results in mild forms of botulism [4].

Four distinct phenotypic groups (I-IV) of *C. botulinum* are recognized. *C. botulinum* Groups I and II are primarily responsible for human botulism, the most frequent form of botulism in the European Union [5]. *C. botulinum* Group I has minimum and optimum growth temperatures of 12 °C and 37 °C, respectively. Spores formed by strains of this group are highly heat-resistant. Treatment of 121 °C for 3 min of low-acid canned foods is required for their inactivation. *Clostridium sporogenes* is often viewed as a non-toxigenic equivalent of *C. botulinum* Group I [6]. *C. botulinum* Group II strains forming type B or type E neurotoxin are most frequently associated with foodborne botulism due to spore survival in the pasteurization process and their ability to germinate and produce neurotoxin at refrigeration temperatures. The minimum growth temperature for this bacterium is 3 °C [7]. The first strain of *C. botulinum* of this type (*C. botulinum* Group II, type B) was isolated by Emilie van Ermengem in 1897, following an outbreak of foodborne botulism involving salted ham in Ellezelles, Belgium [6]. Recent foodborne botulism outbreaks in Iceland, the United Kingdom, and France were also associated with *C. botulinum* Group II, type B [6]. *C. botulinum* group III strains producing neurotoxins types C, D, and chimers C/D and D/C are mainly connected to animal botulism, and *C. botulinum* group IV is a rarer group that produces neurotoxin of type G [8].

The initial symptoms of foodborne botulism are often confused with more common health concerns such as stroke, myasthenia gravis, Eaton–Lambert syndrome, or tick paralysis [5]. The treatment includes gastrointestinal decontamination, administration of the specific antidote, and, when necessary, respiratory support. Two primary BoNT antitoxins, trivalent (A, B, and E) and heptavalent (A, B, C, D, E, F, and G), can neutralize the free circulating toxins, preventing their binding to the neuromuscular junction [5]. However, there is a growing interest in developing novel strategies to treat botulism. Recently, a new methodology using atoxic derivatives of BoNT to transport therapeutic antibodies into the neuronal cytosol to bind and neutralize BoNT has been developed [9]. Modern technology may enable the delivery of antibodies and other protein-based therapeutics to previously inaccessible intraneuronal targets [10]. Another strategy is using bacteriophage (viruses that infect bacteria) lytic enzymes, called endolysins, to treat and prevent botulism. Typically, endolysins degrade bacterial peptidoglycan at the end of the bacteriophage lytic cycle to facilitate phage progeny release. These enzymes can recognize and digest a specific chemical bond(s) within peptidoglycan and therefore can be classified as *N*-acetylmuramidases (lysozymes), *N*-acetyl-β-d-glucosaminidases (glycosylases), *N*-acetylmuramoyl-l-alanine amidases, l-alanoyl-d-glutamate endopeptidases, and interpeptide bridge-specific endopeptidases [11].

Researchers have long searched for and characterized new endolysins for extracellular use to combat bacterial infections. Until now, several phage endolysins have been shown to exhibit antibacterial activities against different *Clostridium* strains. The most studied examples are endolysins active against *Clostridium perfringens*, the third leading cause of human foodborne illnesses [12]. Ply3626 was the first discovered endolysin of *C. perfringens* bacteriophage [13], and since then, a few others, including PlyCP390 and PlyCP26F [14], Psm-his [15], PlyCM [16], CP25L [17], PlyCP10, PlyCP41 [12], LysCPS2 [18], LysCP2 [19], and LysCPAS15 [11], have been characterized. In general, lytic enzymes of *C. perfringens* phages, like many endolysins of bacteriophages of Gram-positive bacteria, have two domains: the enzymatically active domain (EAD) responsible for the catalytic function of the protein and the cell-wall-binding domain (CBD) that targets the endolysin to the host. Due to the presence of the CBD, which specifically recognizes and noncovalently binds to the ligand molecules within the bacterial cell envelope, *C. perfringens* phage endolysins display a narrow host spectrum limited to the bacteria of the *Clostridium* genus [11]. Recently, several putative endolysins with activity of N-acetylmuramoyl-L-alanine amidases with amidase_2 or amidase_3 catalytic domains have been identified in the genomes of bacteriophages/prophages of *Clostridium difficile* strains [20]. Two of them, CD27L and PlyCD, are active against several *C. difficile* isolates and were characterized in detail [21,22]. Single endolysins targeting *Clostridium tyrobutyricum* (CTP1L), *Clostridium sporogenes* (CS74L), and *C. botulinum* Group I cells (CBO1751) were also reported [23,24,25]. Despite extensive efforts to understand the structure–function relationship of endolysins from *Clostridium* bacteriophages [26,27], there is still little demonstration of their practical applications. No endolysin of bacteriophage of *C. botulinum* Group II has been described so far.

Recent studies in our laboratory resulted in the discovery of two thermostable endolysins, Ph2119 (GenBank accession no. AHF20915.1) and Ts2631 (AIM47292.1), with amino acid sequence similarity to eukaryotic peptidoglycan recognition proteins (PGRPs) [28,29,30]. Both enzymes, derived from *Thermus scotoductus* bacteriophages, Ph2119 and vB_Tsc2631, show strong muralytic activity against bacteria of genus *Thermus*. Interestingly, they were also active against mesophilic Gram-negative bacteria such as *Escherichia coli*, *Serratia marcescens*, *Pseudomonas fluorescens*, and *Salmonella enterica* serovar Panama. On the other hand, they were not active against mesophilic Gram-positive bacteria, except for *Bacillus cereus* [28,29]. Recently, our group demonstrated the antibacterial activity of Ts2631 endolysin against *Acinetobacter baumannii* and *Pseudomonas aeruginosa* [31]. Both bacteria are included on the World Health Organization (WHO) list of antibiotic-resistant “priority pathogens” for which new antibiotics are urgently needed [32].

Here, in search of novel antibacterial agents, we performed bioinformatics analysis to identify lytic enzymes similar to thermostable Ph2119 and Ts2631 endolysins. We identified and characterized a novel endolysin named LysB from the prophage of *C. botulinum* E3 strain Alaska E43. The predicted endolysin was overexpressed in *Escherichia coli* Tuner(DE3) and exhibited lytic activity against bacteria of *Clostridium* genus, *Deinococcus radiodurans,* and *Staphylococcus aureus*. No lysis of Gram-negative bacteria was observed. Size-exclusion chromatography and analytical ultracentrifugation showed that the protein is dimeric in solution. LysB can bind to lipoteichoic acids, a significant constituent of the Gram-positive bacterial cell wall, and in silico analysis showed that twelve residues (M13, H43, N47, G48 W49, A50, L73, A75, H76, Q78, N81, and Y182) might be involved in this interaction.

## 2. Results

### 2.1. In Silico Analysis in Search of Lytic Enzymes

**Figure 1:**
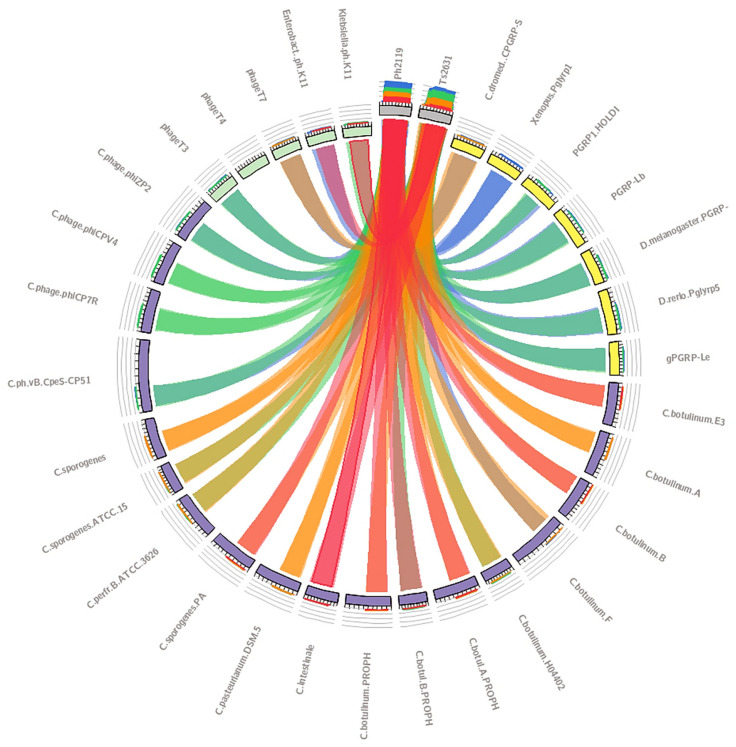
The similarity analysis of thermostable endolysins Ph2119 and Ts2631 compared to phage and bacterial lytic enzymes including *Clostridium* enzymes and eukaryotic proteins recognizing peptidoglycan (PGRPs), visualized using Circoletto software [33]. The Basic Local Alignment Search Tool (BLAST) sequence comparison results are represented in four quartiles, each shown in a different color pattern. Red ribbons reflect the highest score, corresponding to 33–34% amino acid sequence identity, orange and green indicate medium scores, and blue indicates the lowest percentage of identity (26%). The width of the ribbons represents alignment length. Original dataset with the protein amino acid sequences and their respective GenBank or Protein Data Bank (PDB) accessions numbers and the BLAST results with E values are available as Supplementary Materials S1 and S2.

BLASTP computational analysis revealed that thermostable Ph2119 and Ts2631 endolysins show homology not only to PGRP proteins and a putative lytic enzyme from Thermus thermophilus bacteriophage PhiKo (AYJ74695.1), but also to several hypothetical, annotated N-acetylmuramoyl-l-alanine amidases (EC 3.5.1.28), which are lytic enzymes of different Clostridium species (C. perfringens, C. sporogenes, C. pasteurianum, C. intestinale, and C. botulinum) (Figure 1). Among them, there was a putative lytic protein of 224 amino acids encoded in the genome of C. botulinum strain E3 Alaska E43 (GenBank CP001078.1), designated as LysB (GenBank ACD52487).

We have selected this protein for further study, since the recent rise in the emergence of multidrug-resistant virulent Clostridium strains underpins the necessity of developing novel therapeutic strategies [34]. The availability of the biosafety level 1 C. sporogenes strain that is often used as a surrogate for C. botulinum and does not produce the botulinum neurotoxins [35] also favours our selection.

**Figure 2:**
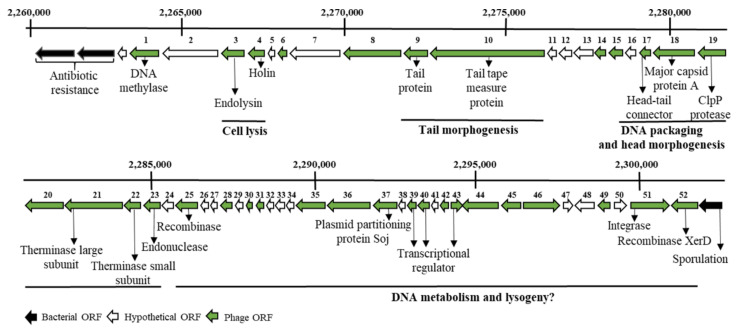
A fragment of *C. botulinum* E3 strain Alaska E43 genome map with predicted open reading frames (ORFs) within the prophage region. ORFs are depicted as arrows in the expected direction of transcription. Gray: bacterial ORF, green: phage ORF, white: hypothetical ORF. Putative functions were assigned based on conserved domain searches. The scheme was performed based on a graphical view of the complete genome sequence of *C. botulinum* E3 strain Alaska E43 (https://www.ncbi.nlm.nih.gov/nuccore/CP001078.1?report=graph; Accessed on 25 May 2021).

Analysis of the complete genomic sequence of the *C. botulinum* strain E3 Alaska E43 with the prophage prediction tool PHASTER revealed the presence of the prophage region. Despite its relatively small genome size of 47,6 kb, the putative prophage showed the overall modular organization following bacteriophages of the *Siphoviridae* family. It consisted of lysogeny, DNA metabolism, DNA packing, head morphogenesis, tail morphogenesis, and host cell lysis modules [36,37,38]. Putative functions of open reading frames (ORFs) were assigned based on conserved domain searches (https://pfam.xfam.org/) (Table S2). In the group responsible for host lysis, there were two interesting ORFs. The first ORF, 675-bp, encoded an endolysin, an *N*-acetylmuramoyl-l-alanine amidase (LysB) of 224 amino acids, and the second ORF, 417-bp, encoded a holin, a protein of 138 amino acids, which triggers cell lysis (Figure 2).

**Figure 3:**
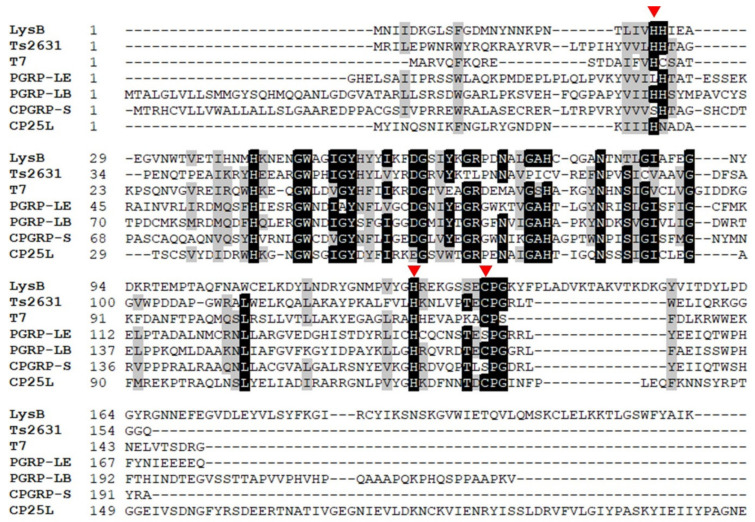
Multiple alignment of the amino acid sequence of LysB endolysin shows its homology to Ts2631 endolysin, T7 lysozyme, and eukaryotic peptidoglycan recognition proteins (PGRPs). GenBank or PDB accession numbers are LysB (ACD52487), Ts2631 from *T. scotoductus* phage vB_Tsc2631 (AIM47292.1), T7 from enterobacteria phage T7 (AAB32819.1), gPGRP-LE from *D. melanogaster* (PDB entry: 2CB3_B), PGRP-LB from *D. melanogaster* (PDB entry: 1OHT_A), CPGRP-S from *C. dromedarius* (PDB entry: 3O4K), CP25L from *C. perfringens* phage vB_CpeS-CP51 (AGH27916.1). The red triangles indicate residues responsible for Zn^2+^ binding (H25, H126, C134). The alignment was generated with the Clustal Omega program with default options. The black background represents 100% amino acid sequence identity, while the gray background indicates amino acid conservation at 70%.

Conserved Domain Database [39] and Pfam [40] analysis assigned to the LysB endolysin an *N*-acetylmuramoyl-l-alanine amidase domain of the amidase_2 class, with a Zn^2+^ catalytic site (Pfam01510). In T7 lysozyme [41] and Ts2631 endolysin [29], the Zn^2+^-coordination site consists of two histidines and one cysteine. In T7 lysozyme, additional tyrosine is bound to Zn^2+^ through a water molecule [41]. Despite the fact that the amino acid sequence of LysB shows only 31.67% identity to Ts2631 endolysin (E value 7 × 10^−16^) and 31.75% identity to T7 lysozyme (E value = 1 × 10^−17^), the Zn^2+^ catalytic triad (H25, H126, C134) is conserved in the LysB endolysin sequence (Figure 3). In addition, the enzyme shares 31.48% sequence identity to gPGRP-LE from *Drosophila melanogaster* (E value = 3 × 10^−13^), 32.6% to PGRP-LB from the same species (E value = 5 × 10^−17^), and 33.7% to CPGRP-S from *Camelus dromedarius* (E value = 3 × 10^−12^). PGRPs are innate immunity proteins prevalent in insects, mollusks, echinoderms, and vertebrates. They contain a conserved *C*-terminal PGRP domain that is homologous to bacteriophage and bacterial type 2 amidases. PGRPs recognize bacterial peptidoglycan and, in some cases, may hydrolyze it, leading to bacterial cell death [42].

### 2.2. Expression and Purification of LysB Endolysin

**Figure 4:**
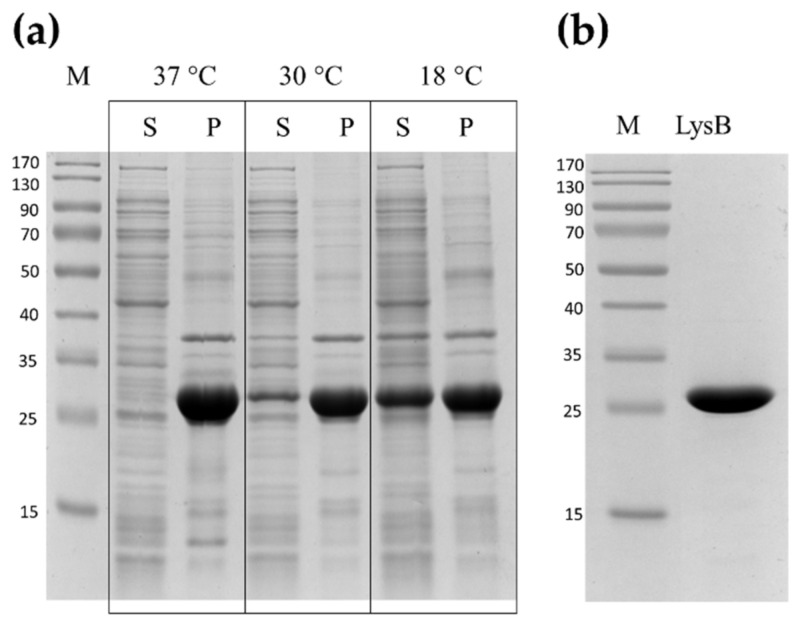
Overproduction and purification of LysB endolysin. (**a**) Overproduction in *E. coli* Tuner(DE3) cells was carried out with 1 mM IPTG for 4 h at both 37 °C and 30 °C or overnight at 18 °C. Then cells were harvested, and after sonication, cell lysates were centrifuged to obtain clear supernatant with soluble proteins (S) and pellet (P) fractions. (**b**) Purified LysB endolysin. All samples were mixed with Laemmli buffer and loaded on 12.5% SDS-PAGE. Gels were stained with Coomassie Brilliant Blue R-250. (M) Protein standards where values at left indicate molecular masses (in kDa) (Thermo Fisher Scientific, Waltham, MA USA).

The gene sequence (675-bp) encoding LysB endolysin was codon-optimized for *E. coli* codon usage. The amplified gene was cloned into expression vector pET15b to construct a pET_LysB recombinant plasmid (with a 6× His tag for ease of purification). First trials to purify the LysB endolysin after standard overproduction at 37 °C showed that after sonication, the protein was present only in an insoluble protein fraction (P) (Figure 4a). Therefore, it was decided to lower the temperature during the LysB endolysin overproduction, and after the induction step, incubation was carried out for 4 h at 30 °C or overnight at 18 °C. The overproduction of LysB at 18 °C resulted in the presence of LysB (seen in Figure 4a) in the supernatant (S), in an amount sufficient to conduct further purification steps. The LysB endolysin was purified using immobilized metal affinity chromatography (IMAC) as outlined under Materials and Methods. The purification yield from 1 L of *E. coli* Tuner(DE3) (pET15b_LysB) culture was 37.5 mg, with a final purified protein concentration of 15 mg/mL achieved. The LysB endolysin with *N*-terminal hexahistidine tag (His-tag) had a predicted molecular weight of 27,726 and isoelectric point of 7.09 (as evaluated by the IPC tool), which corresponded well to the protein size determined by the SDS-PAGE (Figure 4b).

### 2.3. Bacteriolytic Spectrum of LysB Endolysin

**Figure 5:**
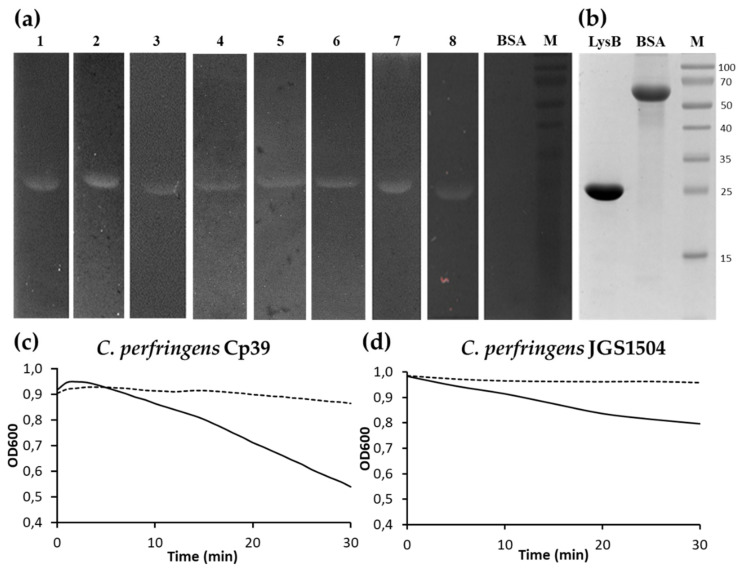
Lytic activity of LysB endolysin. (**a**) Zymogram containing (1) *C. sporogenes* ATCC 7955, (2) *C. intestinale* ATCC 49213, (3) *B. cereus* ATCC 13061, (4) *B. megaterium* ATCC 14581, (5) *B. mycoides* KPD 15, (6) *B. thuringensis* KPD 114, (7) *S. aureus* ATCC 25923, (8) *D. radiodurans* ATCC 13939, and bovine serum albumin (BSA). (**b**) SDS-PAGE (12.5%) analysis of purified LysB endolysin (LysB) and BSA as a quantity control; results of turbidity reduction assay against (**c**) *C. perfringens* Cp39 and (**d**) *C. perfringens* JGS1504. The dotted lines indicate controls (cells without endolysin), and solid lines indicate bacterial cells incubated at 22 °C with LysB at a concentration of 100 µg/mL. The data shown represent one of three independent experiments. (M) Protein standards where values at right indicate molecular masses (in kDa) (Thermo Fisher Scientific, Waltham, MA, USA).

The bacteriolytic activity of LysB endolysin was tested against several Gram-positive and Gram-negative bacterial strains (Figure 5a). In the zymogram assay, LysB was active against *C. sporogenes* ATCC 7955, *Clostridium intestinale* ATCC 49213, *Bacillus cereus* ATCC 13061, *Bacillus megaterium* ATCC 14581, *Bacillus mycoides* KPD 15, *Bacillus thuringensis* KPD 114, *S. aureus* ATCC 25923, and *D. radiodurans* ATCC 13939. The lysis is shown as a white band on the dark background. No lysis was observed in the case of bovine serum albumin (BSA), which served as a negative control (Figure 5b). Endolysin did not show activity against *Bacillus pumilus* KPD 181, *Bacillus subtilis* ATCC 6633, *E. coli* MG1655, *Listeria monocytogenes* KPD 1326, *Micrococcus luteus* ATCC 7468, *Streptococcus pyogenes* KPD 457, *S. enterica* serovar Panama KPD 101, or *Thermus flavus* MAT 1087 (Summary in Table S3). In addition, turbidity reduction assays of LysB endolysin against *C. perfringens* Cp39 and *C. perfringens* JGS1504 under conditions specified in the Materials and Methods section showed activity of the protein against *C. perfringens* Cp39 strain (Figure 5c) and moderate activity against *C. perfringens* JGS1504 (Figure 5d).

### 2.4. Optimal Conditions for LysB activity

**Figure 6:**
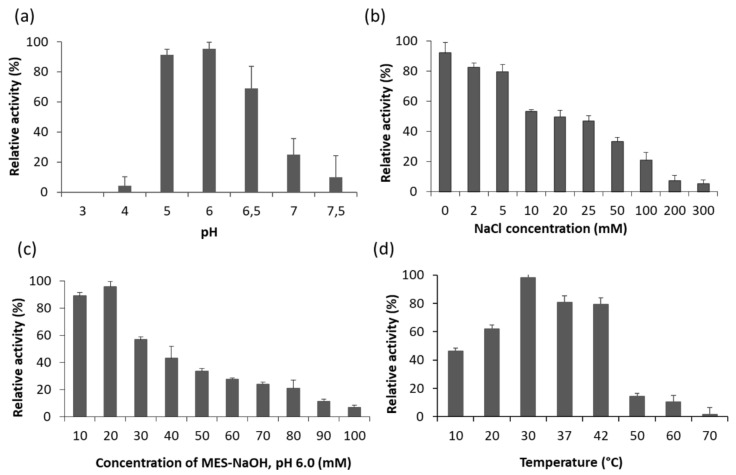
Effects of (**a**) pH; (**b**) NaCl; **(c)** the optimal value of the buffer concentration; and **(d)** temperature on the lytic activity of LysB against *C. sporogenes* ATCC 7955 cells. Relative activity was calculated by comparing the lytic activity at a specific condition with the maximal lytic activity within the dataset. Each experiment was repeated in triplicate, error bars indicate the standard deviations.

The enzyme lytic activity was examined under different conditions using turbidity reduction assay and *C. sporogenes* ATCC 7955 cells as a substrate. The LysB endolysin showed the highest activity at pH 6.0, significantly reduced below pH 5.0 and above pH 6.5 (Figure 6a). At pH 4.0 and 7.0, the activity was reduced to 4.3% and 24.9%, respectively, compared to the maximal lytic activity at pH 6.0. The effect of ionic strength on the functionality of LysB endolysin was estimated at concentrations of NaCl ranging from 0 to 300 mM (Figure 6b) and concentrations of buffer MES-NaOH, pH 6.0, between 10 and 100 mM (Figure 6c). The highest lytic activity was observed without salt added and when 20 mM MES-NaOH, pH 6.0, was used as a reaction buffer. The presence of 10 mM NaCl was sufficient to drop activity to 53.2%, and the gradual increase in salt concentration resulted in a further decrease in LysB endolysin functionality (which was 5.8% at 300 mM NaCl, relative to the highest enzyme activity in the absence of salt). Therefore, further experiments were performed in 20 mM MES-NaOH buffer, pH 6.0, with no NaCl added. The optimal temperature for LysB activity was 30 °C (Figure 6d). The enzyme showed 79.2% of activity at 42 °C, but the further increase in the assay temperature caused a drop in LysB activity to less than 15% (14.3% at 50 °C).

### 2.5. Electron Microscopy Experiments

**Figure 7:**
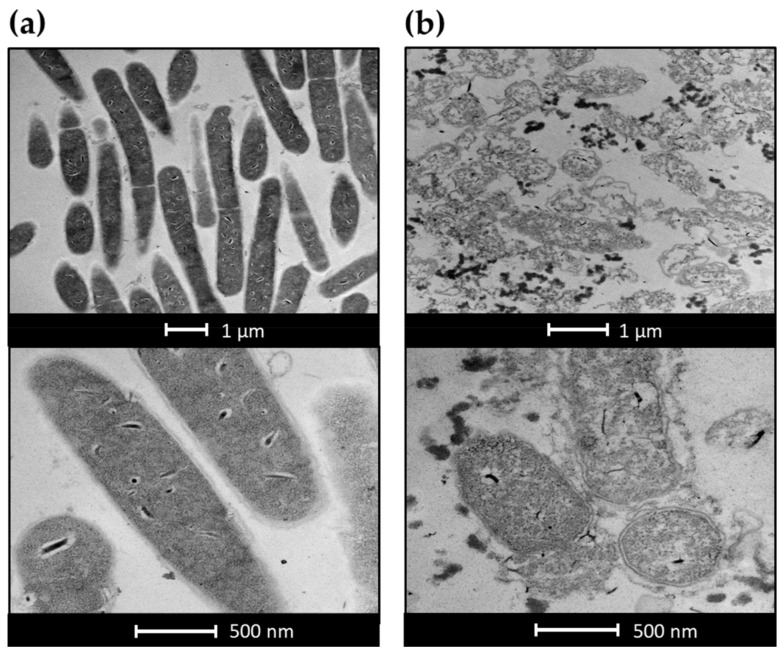
Transmission electron microscopy of LysB-treated *C. sporogenes* ATCC 7955. (**a**) Untreated *C. sporogenes* cells, (**b**) *C. sporogenes* treated with 50 µg/mL LysB for 20 min.

The effects of LysB endolysin activity on the morphology of *C. sporogenes* ATCC 7955 cells were visualized using transmission electron microscopy (TEM). In the control experiment, the cell shape of the untreated bacteria remained unaltered (Figure 7a). The exposure of the bacteria to LysB at a concentration of 50 μg/mL for 20 min caused significant changes in the shape of the cells. The bacteria exposed to LysB endolysin exhibited many abnormalities in morphology, including disruption of the cell wall, detachment of cellular membrane, an outflow of cellular content, and cellular disintegration (Figure 7b).

### 2.6. Oligomeric State of LysB Endolysin

**Figure 8:**
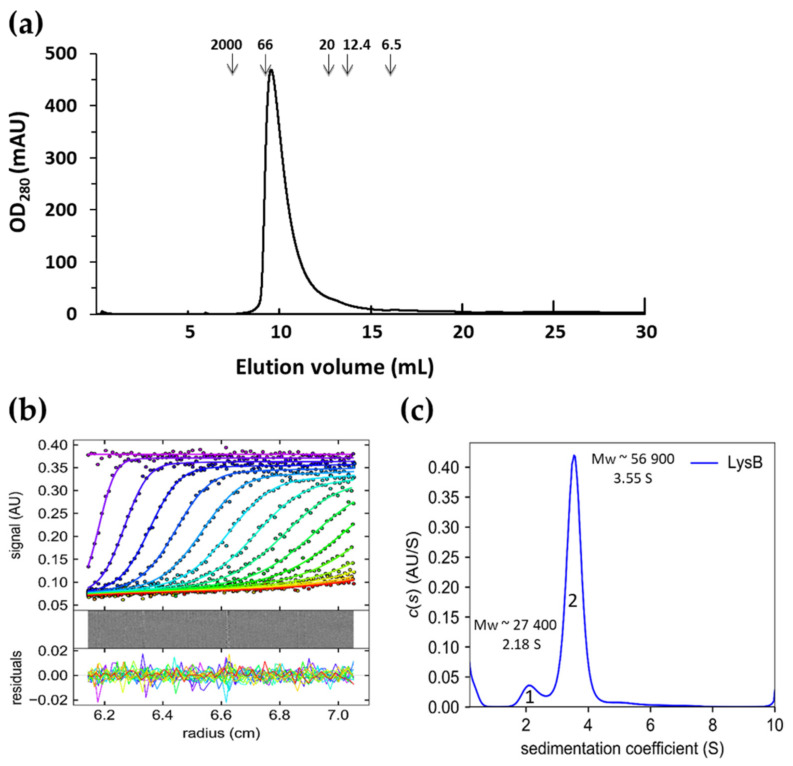
Size-exclusion chromatography and sedimentation velocity analytical ultracentrifugation analysis of LysB. (**a**) Elution profile of LysB endolysin on Superdex 75 sizing column. LysB endolysin (3 mg/mL) in a final volume of 300 µL was loaded on Superdex 75 10/300 GL (AKTA Pure chromatography system, GE Healthcare, Little Chalfont, UK) and eluted at a flow rate of 0.8 mL/min in buffer consisting of 25 mM NaH_2_PO_4_, 150 mM NaCl, pH 8.0. Elution of LysB endolysin was detected by absorption at 280 nm (mAU, milli-absorbance units). The position of molecular mass standards are shown as arrows: dextran blue: 2000 kDa, bovine serum albumin: 66 kDa, trypsin inhibitor: 20 kDa, cytochrome C: 12.4 kDa, aprotinin: 6.5 kDa. (**b**) Sedimentation velocity data were overlaid with the best-fit curves (lines) obtained from sedimentation coefficient distribution analysis. For the clarity of the drawing, only every fifth scan and fifth data point are shown. Below, residuals of the experimental fits. (**c**) Sedimentation coefficient distributions (c(s)) of LysB endolysin. Peak 1 corresponds to LysB monomer; peak 2 corresponds to LysB homodimer.

To determine the oligomeric state of LysB endolysin, the purified protein sample was subjected to size-exclusion chromatography (SEC) and analytical ultracentrifugation (AUC). The results are shown in Figure 8. As a reference, SEC of the control’s dextran blue (2000 kDa), bovine serum albumin (66 kDa), trypsin inhibitor (20 kDa), cytochrome C (12.4 kDa), and aprotinin (6.5 kDa) was performed. The elution profile of the LysB protein revealed a single peak with an elution volume of 9.55 mL that corresponds to a molecular weight of 60,000 (Figure 8a). This SEC result suggests that the protein exists in a dimeric form (homodimer) in solution. The analysis also indicated high homogeneity of the protein, suggesting a lack of aggregation. The AUC experiments further supported these results, where by nonlinear fittings, average molecular weights of LysB endolysin were determined as 27,400 (monomer) and 56,900 (dimer) (Figure 8b,c). The experimentally determined molecular weight (MW) of LysB monomer (peak 1, Figure 8c) corresponds well with the calculated MW based on the protein sequence (M*_r_*= 27,726). Moreover, the results of both size-exclusion chromatography and analytical ultracentrifugation agree that in solution, LysB endolysin exists predominantly as a homodimer.

### 2.7. Interaction between LysB and Lipoteichoic Acid

**Figure 9:**
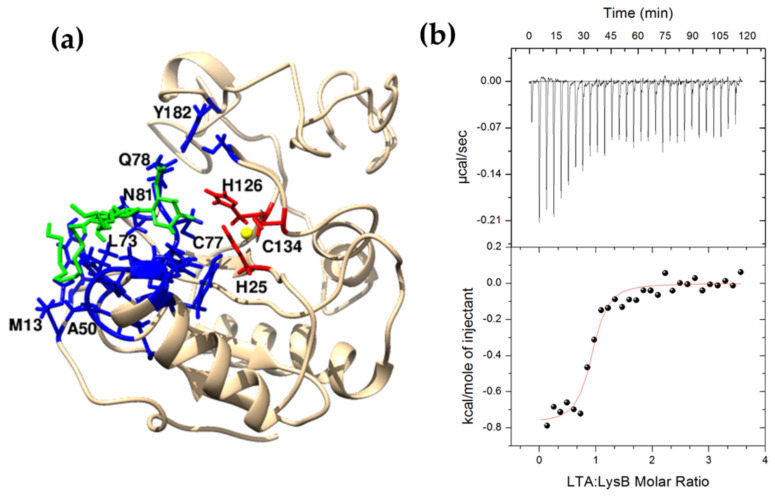
Interaction between LysB and lipoteichoic acid. (**a**) Structural model of LysB endolysin (template: PDB 3O4K). The residues M13, H43, N47, G48 W49, A50, L73, A75, H76, Q78, N81, and Y182 form the LTA binding site (3.5 Å blue zone from green LTA). H25, H126, and C 134 (red) bind Zn^2+^ ion (yellow). For the clarity of the presentation, only one molecule from the LysB homodimer is shown. (**b**) Calorimetric titration isotherm of the binding interaction between LTA and LysB in 20 mM potassium phosphate buffer, pH 8.0, 10% glycerol at 25 °C.

Eukaryotic PGRPs and bacteriophage endolysins, apart from binding to peptidoglycan, may interact with other components of bacterial cell wall such as wall teichoic acids (WTAs) and membrane-anchored lipoteichoic acids (LTAs) [43,44]. The CBD of *Listeria* bacteriophage endolysin PlyP35 interacts with *N*-acetylglucosamine residues in WTAs [44], while the camel PGRP-S (CPGRP-S) binds to *S. aureus* LTAs [43]. In silico comparative analysis showed that LysB endolysin has conserved residues responsible for LTAs binding. A molecular model of the LysB endolysin based on the short form of camel PGRP (CPGRP-S) structure (PDB entry: 3O4K) illustrates LTA binding sites (M13, H43, N47, G48 W49, A50, L73, A75, H76, Q78, N81, and Y182); for more details, see Figure 9a. A representative binding isotherm for *S. aureus* LTA-LysB interactions in 20 mM potassium phosphate buffer, pH 8.0, 10% glycerol at 25 °C, is shown in Figure 9b. The thermodynamic parameters, namely binding constant (log *K*_ITC_ = 6.15 ± 0.02 M^−1^) and the enthalpy change (Δ*H*_ITC_ = −0.77 ± 0.02 kcal mol^−1^), were obtained directly from ITC measurements by fitting isotherms (using nonlinear least-squares procedures) to a model that assumes one set of binding sites. The assumed model yields the best fit of calculated vs. experimental data. The standard thermodynamic relationships were as follows: Δ*G*_ITC_ = -RTln*K*_ITC_ = Δ*H*_ITC_ − TΔ*S*_ITC_ was used to calculate the free energy of binding (Δ*G*_ITC_ = −8.41±0.02 kcal mol^−1^) and the entropy change, (TΔ*S*_ITC_ = 7.64 ± 0.03 kcal mol^−1^).

It is noteworthy that according to the bioinformatics analysis, the LysB catalytic domain appears to have a dual function, able to both degrade peptidoglycan and dock the protein to the bacterial cell wall. These two functions of LysB seem to be independent, as suggested by the fact that the residues involved in Zn^2+^ coordination and catalysis (H25, H126, and C134) are not engaged in the LTA binding (M13, H43, N47, G48 W49, A50, L73, A75, H76, Q78, N81, and Y182) (Figure 9).

### 2.8. Functional Analysis of the LysB Catalytic Site

Multiple sequence alignment of LysB endolysin and related proteins (Ts2631 endolysin from *T. scotoductus* bacteriophage vB_Tsc2631, T7 lysozyme, PGRP-LE and PGRP-LB from *D. melanogaster,* and CPGRP-S from *C. dromedarius*) revealed conserved residues potentially involved in Zn^2+^ and substrate binding (Figure 3). Previous detailed T7 lysozyme activity studies showed that tyrosine Y46, and lysine K128 residues play a significant role in catalysis [41]. In the amidase-active PGRPs such as PGRP-LB, threonine residue T158, which corresponds to K128 in T7 lysozyme, is highly conserved [45]. In the cases of PGRP-LB and T7 lysozyme, substitutions T158K and K128T, respectively, resulted in a significant decrease in these enzymes’ lytic activity [41,45]. In the LysB endolysin primary sequence, the Y54 residue corresponds to Y46 of T7 lysozyme, and S132 corresponds to K128 of T7 lysozyme and T158 of PGRP-LB. Moreover, three conserved residues (histidines H17 and H122 and cysteine, C130) that form the T7 lysozyme Zn^2+^ binding site correspond to residues H25, H126, and C134 in the LysB enzyme (Figure 4). All three residues are in close proximity to one another in the enzyme tertiary structure (Figure 9a). To investigate the role of Zn^2+^ in the lytic activity of LysB, the enzyme was treated with 5 mM EDTA for 30 min at 30 °C. This treatment abolished the activity of the enzyme (Table 1). After overnight dialysis of the EDTA-treated sample against 20 mM MES-NaOH, pH 6.0, Zn^2+^ cations were added to the protein to achieve a final concentration of 0.1 or 1 mM. The negative control for each sample contained a reaction buffer supplemented with 0.1 mM or 1 mM metal ions. Adding 0.1 mM Zn^2+^ reconstituted the LysB activity to 42.4%, while Zn^2+^ at the 1 mM concentration had no positive effect on the reaction (Table 1).

The involvement of conserved residues in forming the enzyme’s catalytic site was tested using five amino acid substitution variants, namely H25N, Y54F, H126N, S132K, and C134S. They were purified and assayed for lytic activity against *C. sporogenes* ATCC 7955. The lytic activity of the H25N variant was not determined due to protein aggregation. In the cases of other variants, their activity was significantly lower compared to the wild-type protein: Y54F (42%), H126N (37%), S132K (no activity), and C134S (55%) (Table 1). The results obtained indicate the critical role of conserved residues on the lytic activity of LysB endolysin and the requirement of Zn^2+^.

## 3. Discussion

This work presents the cloning, production, and characterization of the LysB lytic enzyme from a putative prophage of *C. botulinum* E3 strain Alaska E43. The location of the *lysB* gene in a prophage region and the presence of a holin gene adjacent to the *lysB* gene (Figure 2, Table S2, positions 6, 7) indicate that the LysB protein is a prophage endolysin and belongs to the second class of murein hydrolases, which are transported through bacterial membranes via holes formed by holins [46]. This is in agreement with the results of the analysis performed by the PrediSi (Prediction of Signal peptides) tool, showing that the LysB enzyme lacks the signal peptide sequence responsible for protein secretion. Comparative analysis showed that LysB endolysin is *N*-acetylmuramoyl-l-alanine amidase (amidase_2 domain) with Zn^2+^ coordination site (Pfam01510). Here, we experimentally confirmed the dependence of LysB lytic activity on the presence of zinc ions (Table 1). Amidase_2 domain with Zn^2+^ in the catalytic center is also present at the *N*-terminal part of CP25L endolysin of *C. perfringens* phage vB_CpeS-CP51 [17], three predicted endolysins from podoviruses of *C. perfringens*, ΦCPV4, ΦZP2, and ΦCP7R [47], and a putative lytic enzyme named CDG from *Peptoclostridium difficile* DA00211 strain [48]. Further analysis showed low LysB amino acid sequence identity (31.67%) to the primary sequence of *Thermus scotoductus* phage Ts2631 endolysin (GenBank accession no. AIM47292.1), 31.75% to T7 lysozyme (AAB32819.1), 32.56% to PGRP-LB (NP_731575.1), and 39% (amino acids 1–172) to CP25L endolysin from *C. perfringens* phage vB_CpeS-CP51 with E value = 4 × 10^−37^ (AGH27916.1). No significant similarity was found between the primary sequence of LysB endolysin and CBO1751 putative amidase from the prophage of *C. botulinum* Group I strain ATCC 3502, which is the only endolysin previously characterized of *C. botulinum* background. The CBO1751 endolysin has an *N*-terminal amidase_3 domain (Pfam01520), with a catalytic site formed by two histidines and two glutamic acids. Bioinformatics analysis revealed that the LysB endolysin active site consists of two histidines: tyrosine and cysteine (Figure 3). The lack of primary sequence similarity between these two endolysins and significant differences in catalytic site organization indicate that LysB is a novel type of endolysin. Its characterization significantly expands knowledge of the endolysins of *Clostridium botulinum* bacteriophages. In contrast to the CBO1751 endolysin, which prefers alkaline pH (from 8.5 to 10.5) for lytic activity, LysB works at pH from 4.0 to 7.5, showing the highest activity at pH 6.0 in 20 mM MES-NaOH. However, this coincides with the minimal growth pH (pH 5.0) of *C. botulinum* group II (botulinum neurotoxin type E) [49], from which the tested endolysin is derived. Furthermore, the pH range for the activity of the LysB endolysin is similar to the CD27L endolysin of *C. difficile* bacteriophage, with amidase_3 domain, which has defined optimal pH range between 4.5 and 8.3 [22]. The optimal pH for activity of LysB endolysin is also similar to other *Clostridium*-targeting endolysins, such as PlyCP10 and PlyCP41, that have maximum activity at pH 6.0 and pH 6.5, respectively [50]. The addition of 10 mM NaCl caused a decrease in the LysB endolysin functionality to 53.2%, while negligible cell lysis (5.8%) was shown at 300 mM NaCl. Previous studies about chimeric PlyGVE2CpCWB endolysin showed that 150 mM NaCl caused a decrease in its activity to 37% [51]; on the other hand, *Clostridium perfringens* phage CPS2 endolysin was highly stable in a wide range of NaCl concentrations [18]. Therefore, there is no specific pattern of dependence between NaCl concentration and lytic activity of bacteriophage endolysins of the genus *Clostridium*. The optimal temperature for LysB lytic activity is 30 °C, which agrees with the psychrotrophilic nature of *C. botulinum* Group II, which has an optimum growth temperature of 30 °C [6].

Lytic enzymes are diverse in respect to substrate specificity. CD11 and CDG amidases of *Peptoclostridium difficile* are highly active against *C. difficile* clinical isolates while ineffective against *Bacillus* or *Staphylococcal* species [48]. Substrate specificity of PlyCP39O and PlyCP26F endolysins from clostridial phages phiCP39O and phiCP26F, respectively, is very narrow and includes only *C. perfringens* strains, as these enzymes did not lyse non-perfringens clostridial isolates. Some enzymes are active against few species, such as PlyCM, which is active against *C. perfringens*, *C. tetani*, *C. septicum,* and *C. beijerinckii* [16]; or Psm-his endolysin, which lyses *C. perfringens* and moderately lyses *C. tetani* and *C. acetobutylicum* [15]. In general, the LysB endolysin acts against three species of *Clostridium* (*C. intestinale*, *C. perfringens,* and *C. sporogenes*) and other Gram-positive bacteria: *B. cereus*, *S. aureus,* and *D. radiodurans* (in zymogram analysis, Figure 5). In this matter, LysB is similar to the CP25L endolysin of *C. perfringens* bacteriophage, which shows activity against several strains of *C. perfringens*, but also against other Gram-positive bacteria such as *B. cereus* and *B. subtilis* [17]. We may speculate that the similarity of catalytic domains of LysB and CP25L enzymes may determine their slightly broader substrate specificity. It could be that they both have binding domains that recognize targets that are more common among Gram-positive bacteria. Endolysins from phages of *Clostridium*, including LysB endolysin, show no activity against Gram-negative bacteria. That may be explained by the presence of the outer membrane as part of Gram-negative bacteria’s cell wall, which is an effective barrier shielding the peptidoglycan layer against the exogenous activity of endolysins [52].

Residues H25, Y54, H126, and C134 of LysB endolysin correspond to residues H17, Y46, H122, and C130 of T7 lysozyme, which is a well-known type 2 N-acetylmuramoyl-L-alanine amidase and can inhibit T7 RNA polymerase. Substitutions of H17 eliminated the amidase activity of T7 lysozyme [41]. Three H17 substitution variants also showed a decreased ability to inhibit T7 RNA polymerase, suggesting that these changes affected the structure of T7 lysozyme more generally [41]. Although not definitive, this is in agreement with our results, as the substitution variant of H25 residue, which corresponds to H17 of T7 lysozyme, was insoluble during purification trials, suggesting problems with protein conformation. All other LysB substitution variants, including Y54F, H126N, and C134S, revealed decreased activity, further suggesting the involvement of these residues in LysB endolysin lytic activity.

Many globular endolysins are present as monomers in the solution. This property was observed in the case of the thermostable Ts2631 endolysin, T7 lysozyme, and the catalytic domain of CD27L of *C. difficile* ΦCD27 phage [26,42,53]. However, the full-length CD27L endolysin with both catalytic and cell wall binding domains forms a dimer in solution similarly to analytical ultracentrifugation results obtained for LysB (Figure 5). The same mechanism of oligomerization as in the case of CD27L applies to the CTP1L endolysin that targets *C. tyrobutyricum* and the CS74L endolysin that targets *C. sporogenes*. There, the dimer is formed between the full-length protein and the *N*-terminally truncated *C*-terminal cell wall binding domain (CBD) [27]. The LysB endolysin does not have a known CBD but may contain a novel CBD that could contribute to dimer formation. A dimeric homology-based model of LysB has been proposed (Supplementary Movie S1), but further studies will be needed to confirm the mechanism of dimerization, which crystallographic studies might facilitate. Crystallography can also prove helpful for detailed analysis of the docking of the LysB endolysin to lipoteichoic acid as predicted bioinformatically. These studies were performed in the case of CPGRP-S from *C. dromedarius*, where the LTA was held inside the complex of four protein molecules [43]. Experimental evidence related to endolysin–LTA interactions is extremely scarce. More is known about LTAs and bacterial autolytic systems. Bacteria produce autolysins that, similarly to endolysins, hydrolyze bonds in peptidoglycan for several important bacterial physiological processes, such as cell division, cell separation, and peptidoglycan maturation. The pneumococcal cell wall autolysin LytC lysozyme specifically targets choline residues present in lipoteichoic acids of *Streptococcus pneumoniae* [54]. Moreover, the LTAs specifically inhibit the activity of another pneumococcal autolytic enzyme, an N-acetylmuramyl-L-alanine amidase of *S. pneumoniae* R36A strain, but the mechanism of this inhibition is unknown [55]. Therefore, the LysB catalytic domain’s interaction with LTAs is unique and not previously encountered in the field of endolysins.

LysB endolysin is the first characterized enzyme from the prophage of *C. botulinum* group II (botulinum neurotoxin type E), lyses clostridia, and other Gram-positive bacteria such as *B. cereus* or *S. aureus*; it is an interesting enzyme for further studies to elicit its full antibacterial potential. It is especially noteworthy that phage endolysins are highly refractory to resistance development, and despite repeated attempts, no strains of host bacteria that can resist the lytic activities of their bacteriophage endolysins have been reported [56,57].

## 4. Materials and Methods

### 4.1. Bacterial Strains and Growth Conditions

*Clostridium sporogenes* ATCC 7955, *Clostridium intestinale* ATCC 49213, *Bacillus cereus* ATCC 13061, *Bacillus megaterium* ATCC 14581, *Bacillus subtilis* ATCC 6633, *Deinococcus radiodurans* ATCC 13939, *Micrococcus luteus* ATCC 7468, and *Staphylococcus aureus* ATCC 25923 were purchased from the American Type Culture Collection. *Thermus flavus* MAT1087 was kindly provided by MATIS collection of microorganisms, Reykjavík, Iceland. *Bacillus mycoides* KPD 15, *Bacillus thuringiensis* KPD 114, *Escherichia coli* MG1655, *Salmonella enterica* serovar Panama KPD 101, *Listeria monocytogenes* KPD 1326, and *Streptococcus pyogenes* KPD 457 came from the KPD, Collection of Plasmids and Microorganisms at the University of Gdańsk, Poland (WDCM 1084). *Clostridium perfringens* Cp39 cells (poultry isolate) and *C. perfringens* JGS1504 (swine isolate) were kindly provided by Steven M. Swift (USDA ARS NEA BARC Animal Biosciences and Biotechnology Laboratory, Beltsville, MD, USA). *C. perfringens* strains were grown anaerobically at 37 °C in BYC medium (37 g/L brain heart infusion, 5 g/L yeast extract, 0.5 g/L l-Cysteine). *C. sporogenes* ATCC 7955 and *C. intestinale* ATCC 49213 were cultivated under anaerobic conditions, and *S. aureus* ATCC 25923 under aerobic conditions at 37 °C in tryptic soy bullion (TSB) (Graso Biotech, Starogard Gdanski, Poland). *T. flavus* was cultivated at 60 °C in TM medium [28]. *L. monocytogenes* KPD 1326 and *S. pyogenes* KPD 457 were grown at 37 °C in Brain Heart Infusion (BHI) (Graso Biotech). All other bacteria were cultivated at 37 °C in Luria-Bertani (LB) broth [58]. When necessary, LB was supplemented with 100 μg/mL of ampicillin. *E. coli* DH5α cells (Thermo Fisher Scientific, Waltham, MA USA were used for molecular cloning and site-directed mutagenesis. *E. coli* Tuner(DE3) cells (Sigma-Aldrich, St. Louis, MO, USA) were used for protein expression. Vector pET15b (Novagen) was used for cloning and overexpression of the gene coding for LysB endolysin.

### 4.2. Computational Analysis and Molecular Modelling

PHAge Search Tool Enhanced Release (PHASTER) [59] was used to predict the presence of a prophage region in the genome of *C. botulinum* E3 strain Alaska E43 (accessible through University of Alberta; http://www.phaster.ca/; accessed on 25 May 2021). The similarity of Ph2119 and Ts2631 enzymes to phage endolysins and peptidoglycan recognition proteins was visualized with Circoletto software [33], available through the Bioinformatics Analysis Team server (http://tools.bat.infspire.org; accessed on 25 May 2021). Protein sequences were aligned using the CLUSTAL Omega program with default options [60], available through the European Bioinformatics Institute website (http://www.ebi.ac.uk; accessed on 25 May 2021). The molecular weight and isoelectric point of the LysB endolysin were predicted using the IPC tool [61]. The three-dimensional structure of LysB endolysin was predicted using homology modeling. A homology model was built using multiple templates (6fhg_A, 6fhg 2xz4_A, 1yb0_B, 4z8i_A, and 1lba_A) with the I-Tasser program [62], and then side chains were refined by DeepRefiner [63]. Dimerization and LTA binding were inferred using the crystal structure of peptidoglycan recognition protein (CPGRP-S) from *Camelus dromedarius* as the main template (PDB entry: 3O4K) and Zn^2+^ located according to *B. subtilis* endolysin (PDB entry: 3HMB). The model was refined using USCF Chimera [64]. The files related to the bioinformatics analysis are available at: https://doi.org/10.18150/NMEJQ6; accessed on 2 August 2021.

### 4.3. DNA Manipulations

Standard procedures were used for molecular cloning [58]. The synthetic *lysB* gene encoding the LysB putative *N*-acetylmuramoyl-l-alanine amidase (GenBank: ACD52487.1) from *C. botulinum* E3 strain Alaska E43 (GenBank: CP001078.1) was purchased from GeneArt Gene Synthesis Service (Life Technologies, Regensburg, Germany) with codons optimized for expression in *E. coli*. The gene was amplified by polymerase chain reaction (PCR) using PrimeSTAR GXL DNA polymerase (Takara Bio Europe AB, Goteborg, Sweden) and LysB_F and LysB_R primers containing NdeI and BamHI restriction sites, respectively (shaded in Table S1). The PCR product was digested with NdeI and BamHI and ligated into vector pET15b, which has the *N*-terminal hexahistidine (His-tag) sequence. Obtained clones were verified by automated DNA sequencing. Plasmid pET15b_LysB was transformed into *E. coli* Tuner (DE3) for recombinant protein expression. Restriction endonucleases and DNA-modifying enzymes were purchased from Thermo Fisher Scientific Inc. Site-directed mutagenesis was utilized to introduce missense mutations in codons of five amino acids, namely His25, Tyr54, His126, Ser132, and Cys134 of the LysB endolysin sequence. The procedure was performed following the QuickChange II Site-Directed Mutagenesis Kit Manual (Agilent Technologies, Santa Clara, CA, USA), using mutagenic primers listed in Table S1. The presence of the correct substitution in all constructs was confirmed by DNA sequencing. The recombinant plasmids carrying the mutated *lysB* gene were introduced into *E. coli* Tuner(DE3) by chemical transformation. Plasmids constructed in this study were deposited in the Collection of Plasmids and Microorganisms, KPD, University of Gdansk, Gdansk, Poland.

### 4.4. Expression and Purification of LysB Endolysin and Its Substitution Variants

*E. coli* Tuner(DE3) cells, harboring plasmid pET15b_LysB or its mutated versions, were grown in LB medium at 37 °C supplemented with ampicillin to an optical density (OD_600_) = 0.5. Production of recombinant proteins was induced by adding isopropyl-β-d-thiogalactopyranoside (IPTG) to a final concentration of 1 µM. In preliminary experiments, production was carried out either overnight at 18 °C or for 4 h at 30 °C or 37 °C. Overnight induction at 18 °C, optimal for recombinant protein production, has been chosen for further analyses. Next, bacteria were harvested by centrifugation (10,000× *g*, 20 min, 4 °C) and suspended in 20 mL of NPi buffer (50 mM NaH_2_PO_4_, pH 8.0, 300 mM NaCl, 10 mM imidazole, 2 mM 2-mercaptoethanol, 0.1% Triton X-100, 10% (*v*/*v*) glycerol, and 1 mM phenylmethylsulfonyl fluoride [PMSF]). Bacteria were disrupted by sonication (30 bursts of 10 s at an amplitude of 12 µm), and after centrifugation, the clear lysate was mixed with 4 mL TALON cobalt metal affinity resin (TAKARA Bio). The suspension was incubated on ice for 20 min with gentle shaking, and the purification procedure was continued according to the manufacturer’s recommendations. The resin was washed with NPi buffer containing 10 mM and 20 mM imidazole, respectively. Elution was conducted with 150 mM imidazole in NPi buffer. The purified proteins were dialyzed overnight into the storage buffer (25 mM potassium phosphate buffer (KPi), pH 8.0, 50 mM KCl, 0.1% Triton X-100, and 60% glycerol) and kept at −20 °C until further use. Bradford assay was applied to determine protein concentration.

### 4.5. Isothermal Titration Calorimetry

All ITC experiments were performed at 25 °C using the AutoITC isothermal titration calorimeter (MicroCal Inc. GE Healthcare, Northampton, MA, USA). The details of the measuring devices and experimental setup were described previously [65]. The reagents, namely LTA of *S. aureus* (Sigma-Aldrich, St. Louis, MO, USA) and LysB, were dissolved directly in buffer consisting of 20 mM potassium phosphate buffer, pH 8.0, 10% glycerol. The experiment consisted of injecting 10.02 μL (29 injections, 2 μL for the first injection only) of 0.5 mM buffered solution of LTA into the reaction cell, which initially contained 0.03 mM buffered solution of LysB. A background titration, consisting of an identical titrant solution but with the buffer solution in the reaction cell only, was removed from each experimental titration. The LTA solution was injected at 4 min intervals. Each injection lasted 20 s. The stirrer speed was kept constant at 300× rpm. The CaCl_2_-EDTA titration was performed to check the apparatus, and the results (stoichiometry, *K*, Δ*H*) were compared with those obtained for the same samples (a test kit) at Malvern Instruments Ltd. (Malvern, UK).

### 4.6. Testing the LysB Endolysin Optimum

For substrate preparation, *C. sporogenes* ATCC 7955 cells were cultivated in TSB at 37 °C in an anaerobic chamber (DG250 Workstation; Don Whitley Scientific Ltd., Bingley, West Yorkshire, UK) in a volume of 1 L until the mid-log phase was reached (OD_600_ between 0.4 and 0.5). The cells were centrifuged, washed, and suspended in 100 mL of 0.85% NaCl. Before performing the tests, bacteria were suspended in 20 mM 2-morpholinoethanesulfonic acid (MES-NaOH), pH 6.0, to reach the optical density (OD_600_) of 0.7-1.0. The reaction mixtures contained 190 µL of *C. sporogenes* ATCC 7955 cells and 10 µL of LysB endolysin at a final concentration of 50 µg/mL. Tests were performed in a 96-well plate format by measuring the OD_600_ of the suspension after 3 h incubation at 25 °C (or temperatures ranging from 10 to 70 °C, when indicated) with the use of EnSpire multimode plate reader (Perkin Elmer, Waltham, MA, USA). The negative control contained 10 µL of 20 mM MES-NaOH pH 6.0 instead of LysB endolysin. All assays were conducted in triplicate. The lytic activity of LysB was calculated as follows: (ΔOD_600_ sample (endolysin added) − ΔOD_600_ (buffer only))/initial OD_600_ [29].

To evaluate the effect of pH on lytic activity, the substrate *C. sporogenes* ATCC 7955 was suspended in: 20 mM sodium acetate, pH 4.0; 20 mM sodium acetate, pH 5.0; 20 mM MES-NaOH, pH 6.0 and pH 6.5; 20 mM KPi, pH 7.0; and 20 mM Tris-HCl, pH 7.5 of OD_600_ = 1.0. The effect of ionic strength on the lytic activity of LysB endolysin was evaluated with the addition of different concentrations of NaCl (0–300 mM) or different concentrations of buffer MES-NaOH, pH 6.0 (10–100 mM). The influence of Zn^2+^ ions on the lytic activity of LysB was tested as described previously [29]. The minor changes were the incubation of the protein with EDTA at 30 °C and usage of 20 mM MES-NaOH, pH 6.0, as a reaction buffer.

### 4.7. Antibacterial Spectrum of LysB Endolysin

A zymogram assay for detection of bacteriolytic activity was carried out as described previously [66]. Briefly, 5 µg of LysB endolysin and bovine serum albumin (BSA), which served as a negative control, were mixed with 2× Laemmli buffer (125 mM Tris-HCl, pH 6.8; 5% SDS; 10% 2-mercaptoethanol; 20% glycerol; and 0.02% bromophenol blue) and loaded on a 12.5% SDS-polyacrylamide gel containing 0.2% (*wt*/*v*) of bacteria (*C. sporogenes* ATCC 7955, *C. intestinale* ATCC 49213, *B. cereus* ATCC 13061, *B. megaterium* ATCC 14581, *B. mycoides* KPD 15, *B. thuringensis* KPD 114, *S. aureus* ATCC 25923, *D. radiodurans* ATCC 13939, *E. coli* MG1655, *M. luteus* ATCC 7468, *S. enterica* serovar Panama KPD 101, and *T. flavus* MAT1087). After electrophoresis, gels were washed for 30 min in distilled water at room temperature. Then, they were transferred to renaturation buffer (20 mM MES-NaOH, pH 6.0, and 0.1% Triton X-100) and incubated with gentle shaking for 16 h at 37 °C. Subsequently, gels were washed with distilled water, stained with 1% methylene blue in 0.01% KOH for 2 h, and destained with distilled water. The lytic activities appeared as white bands on the dark background.

Turbidity reduction assay (TRA) was used to characterize endolysin activity in a solution using the previously described method with some modifications [67]. For this assay, *C. perfringens* (Cp) cultures were grown anaerobically at 37 °C to mid-log phase in BYC medium. Next, the cells were centrifuged, washed three times with sterile distilled water, and resuspended in water to OD_600_ of ~2.0. The assay was run in a 96-well plate with 100 µL of enzyme plus 100 µL of cells mixed in one well, resulting in a starting OD_600_ of ~1.0 for the reaction. The 96-well plate was read in a SpectraMax 340 plate reader (Molecular Devices, LCC, San Jose, CA, USA), with the plate read every 20 s at 22 °C for 30 min. The data were collected and analyzed using SoftMax Pro software (Molecular Devices, LCC, San Jose, CA, USA). LysB enzyme in elution buffer (NPi buffer with 150 mM imidazole) was diluted from a stock concentration of 0.8 mg/mL into the respective assay buffer (25 mM Tris-HCl, pH 8.0; 20 mM KPi buffer, pH 7.5; or 20 mM MES-NaOH, pH 6.0) to make 0.2 mg/mL of the enzyme. When added to an equal volume of cells in water, the final concentration of LysB was 0.1 mg/mL in the assay.

### 4.8. Size-Exclusion Chromatography (SEC) and Analytical Ultracentrifugation (AUC)

SEC analyses were performed using a Superdex 75 10/300 GL column on an AKTA pure 25 chromatography system (GE Healthcare). The column was equilibrated with a buffer consisting of 25 mM NaH_2_PO_4_ and 150 mM NaCl, pH 8.0, and loaded with 300 µL of LysB endolysin (3 mg/mL). The flow rate was set to 0.8 mL/min, and the absorbance was measured at 280 nm (mAU, milli-absorbance units). As a reference, the column was loaded with 300 µL of dextran blue, 2 mg/mL (2000 kDa); bovine serum albumin, 5 mg/mL (66 kDa); trypsin inhibitor, 2 mg/mL (20 kDa); cytochrome C, 2 mg/mL (12.4 kDa); and aprotinin, 3 mg/mL (6.5 kDa).

Sedimentation velocity experiments were performed in a ProteomeLab XL-I analytical ultracentrifuge (Beckman-Coulter, Inc., Brea, CA, USA), equipped with AN 60Ti 4-hole rotor, 12 mm path length, and double-sector charcoal-epon cells, and loaded with 400 μL of LysB (3 mg/mL) and 410 μL of buffer (25 mM KPi buffer, pH 8.0, 50 mM KCl). The experiments were carried out at 20 °C and 50,000× rpm, using continuous scan mode and radial spacing of 0.003 cm. Scans were collected in 4 min intervals at 280 nm. The fitting of absorbance versus cell radius data was performed using SEDFIT software, version 16.1 [68], and the continuous sedimentation coefficient distribution c(s) model, covering the range of 0–10 S. The confidence level (F-ratio) was specified to 0.68. The frictional ratio parameter f/f0 = 1.47 was calculated as part of the non-linear regression fit. Biophysical parameters of the buffer, density (1.00825 g/mL), and viscosity (0.01018 P) at 20 °C were measured using Anton Paar DMA 5000 density meter and Lovis 2000 ME viscometer. Proteins’ partial specific volumes (V-bars) were estimated using SEDNTERP software (version 1.09, http://www.jphilo.mailway.com/download.htm; accessed on 25 May 2021). The results were plotted using the GUSSI program (version 1.4.1, Chad Brautigam, http://biophysics.swmed.edu/MBR/software.html; accessed on 25 May 2021).

### 4.9. Transmission Electron Microscopy

The *C. sporogenes* ATCC 7955 cells were cultivated in 10 mL of TSB at 37 °C under anaerobic conditions until an exponential growth phase was reached. The cells were centrifuged, washed, and resuspended in 20 mM MES-NaOH, pH 6.0, to reach ~10^7^ cells in a volume of 500 µL. The bacteria were incubated under anaerobic conditions at 37 °C for 20 min with LysB endolysin at a final concentration of 50 µg/mL. The negative control contained buffer (25 mM MES-NaOH, pH 6.0) instead of the LysB endolysin. Bacteria were washed twice with PBS; then, the pellet was fixed with 2.5% glutaraldehyde (Polysciences Inc., Warrington, PA, USA) and post-fixed with 1% osmium tetroxide (Polysciences Inc.). Bacteria were dehydrated with ethanol and embedded in Epon 812 resin (Sigma-Aldrich, St. Louis, MO, USA) Ultrathin sections were prepared with Leica UC7 ultramicrotome (60 nm). Sections were stained with lead citrate and uranyl acetate. Bacterial cells were studied at 120 kV using the Tecnai Spirit BioTWIN electron microscope (FEI Company, Hillsboro, OR, USA).

## Figures and Tables

**Table 1:** Relative lytic activity of LysB variants. The activity was measured using turbidity reduction assay and *C. sporogenes* ATCC 7955 cells as a substrate. ^a^ Lytic activity of EDTA-treated LysB. ^b^ Lytic activity of EDTA-treated and -dialyzed LysB against chloroform-treated *C. sporogenes* ATCC 7955 cells supplemented with Zn^2+^ metal ions at 0.1 mM concentration. ^c^ The reaction conditions were the same as in sample ^b^, but Zn^2+^ was supplemented to 1 mM concentration. Nd—the activity of variant H25N was not determined because it remained insoluble during purification.

Variants	Relative Activity (%)
LysB	100.0 ± 4.4
LysB + 5 mM EDTA ^a^	0.0
LysB + 0.1 mM Zn^2+ b^	42.4 ± 1.8
LysB + 1 mM Zn^2+ c^	0.0
H25N	Nd
Y54F	42.0 ± 4.4
H126N	37.0 ± 2.9
S132K	0.0
C134S	55.0 ± 9.4

## Data Availability

The files related to the bioinformatics analysis are available at: https://doi.org/10.18150/NMEJQ6.

## Supplementary Materials

The following are available online at https://www.mdpi.com/article/10.3390/ijms22179536/s1: Table S1: Sequences of PCR primers used in this study; Table S2: Analysis of the part of genome *C. botulinum* E3 strain Alaska E43 by the prophage prediction program PHASTER; Table S3: Summary of lytic activity of LysB endolysin against representative bacterial strains; Supplementary File S1: Protein amino acid sequences and their respective GenBank or PDB accessions numbers; Supplementary File S2: BLAST results for Circoletto analysis.
