# Supplementary material for: Novel Lytic Enzyme of Prophage Origin from Clostridium botulinum E3 Strain Alaska E43 with Bactericidal Activity against Clostridial Cells
Source: Int J Mol Sci. 2021 Sep 2;22(17):9536. doi: 10.3390/ijms22179536 (PMC8430805; doi:10.3390/ijms22179536)
Supplement: Supplementary file 1 [file ijms-22-09536-s001.zip › ijms-1255678 supplementary tables s1-3.pdf]

International Journal of *Molecular Sciences*

MDPI

Supplementary Materials

Table S1. Sequences of PCR primers used in this study.

Primer Names	Primer Sequences (5`–3`)
	**Cloning of *lysB* gene**
LysB_F	CAGGCGCAT ATGAACATCATCGATA
LysB_R	GTCTACGGA TCCTTATTTGATGGCA
	**Site-directed mutagenesis of *lysB* gene**
H25N_F	AACACCCTGATCGTGAATCACATTGAAGCAGAA
H25N_R	TTCTGCTTCAATGTGATTCACGATCAGGGTGTT
Y54F_F	TGGGCAGGCATCGGCTTTCACTATTATATCAAA
Y54F_R	TTTGATATAATAGTGAAAGCCGATGCCTGCCCA
H126N_F	ATGCCGGTTTATGGTAATCGTGAAAAAGGTAGC
H126N_R	GCTACCTTTTTCACGATTACCATAAACCGGCAT
S132K_F	CGTGAAAAAGGTAGCAAGGAATGTCCGGGTAAA
S132K_R	TTTACCCGGACATTCCTTGCTACCTTTTTCACG
C134S_F	GGTAGCAGCGAAAGTCCGGGTAAATACTTTCCG
C134S_R	CGGAAAGTATTTACCCGGACTTTCGCTGCTACC

The shaded bases indicate restriction enzyme sites (NdeI and BamHI, respectively). The underlined bases indicate substitution sites (H25N,Y54F, H126N, S132K and C134S).

Table S2. Analysis of the part of genome *C. botulinum* E3 strain Alaska E43 by the prophage prediction program PHASTER.

ORF	Location	Size (aa)	BLAST Hit (E-value)	Domain(s)
	Complement 2260969-2262159	396	*Clostridium botulinum*; D-alanyl-D-alanine carboxypeptidase (0.0)	COG1686, D-alanyl-D-alanine carboxypeptidase; penicillin-binding protein 5
	Complement 2262897-2263979	360	*Clostridium botulinum*; MBL fold metallo-hydrolase (0.0)	COG2333, metal-dependent hydrolase, beta-lactamase superfamily; pfam00753, metallo-beta-lactamase superfamily
	Complement 2264191-2264412	73	Hypothetical protein (0.0)	
1	Complement 2264698-2265576	292	PHAGE_Parame_1; DNA methylase (2e-12)	Pfam02086, D12 class N6 adenine-specific DNA methyltransferase
2	Complement 2265638-2267227	529	Hypothetical protein (0.0)	Pfam13175, AAA ATPase domain
3	Complement 2267480-2268154	224	PHAGE_Clostr_vB_CpeS_CP51_NC_021325: endolysin (2.34e-31)	Cd06583, peptidoglycan recognition proteins (PGRPs) are pattern recognition receptors that bind; pfam01510, N-acetylmuramoyl-L-alanine amidase
4	Complement 2268216-2268632	138	PHAGE_Clostr_vB_CpeS_CP51_NC_021325: putative holin (2.64e-62)	Pfam05105, bacteriophage holin family; TIGR01593, toxin secretion/phage lysis holin
5	Complement 2268732-2268956	74	Hypothetical protein (0.0)	
6	Complement 2269044-2269358	104	PHAGE_Paenib_phiIBB_Pl23_NC_021865: hypothetical protein (4.78e-05)	
7	Complement 2269372-2270889	505	hypothetical protein (0.0)	
8	Complement 2270908-2272653	581	PHAGE_Lactob_PLE2_NC_031036: hypothetical protein (2.99e-31)	COG4926, phage-related protein; pfam06605, prophage endopeptidase tail; TIGR01665, phage minor structural protein

9	Complement 2272650-2273324	224	PHAGE_Clostr_phiCT19406C_NC_029006: tail protein (9.88e-19)	COG4722, phage-related protein; pfam05709, phage tail protein; TIGR01633, putative phage tail component
10	Complement 2273340-2276810	1156	PHAGE_Clostr_phiCP26F_NC_019496: phage tail tape measure protein (5.41e-84)	TIGR01760, tail tape measure protein; pfam10145, phage-related minor tail protein
11	Complement 2276860-2277123	87	hypothetical protein (0.0)	
12	Complement 2277126-2277449	107	hypothetical protein (0.0)	
13	Complement 2277452-2278033	193	hypothetical protein (0.0)	
14	Complement 2278036-2278356	106	PHAGE_Lactoc_bIL285_NC_002666: Orf49 (3.35e-14)	
15	Complement 2278349-2278738	129	PHAGE_Strept_phiARI0131_2_NC_031941: hypothetical protein (8.17e-16)	
16	Complement 2278738-2279073	111	conserved hypothetical protein (0.0)	TIGR01563, phage head-tail adaptor
17	Complement 2279048-2279377	109	PHAGE_Lister_LP_030_2_NC_021539: putative head-tail connector protein (9.05e-17)	
18	Complement 2279392-2280588	398	PHAGE_Clostr_phiCD6356_NC_015262: putative major capsid protein A (2.56e-87)	TIGR01554, phage major capsid protein; pfam05065, phage capsid family
19	Complement 2280588-2281379	263	PHAGE_Paenib_HB10c2_NC_028758: ATP-dependent Clp protease proteolytic subunit (1.52e-50)	Cd07016, caseinolytic protease (ClpP) is an ATP-dependent
20	Complement 2281381-2282529	382	PHAGE_Bacill_vB_BhaS_171_NC_030904: hypothetical protein (2.17e-59)	Pfam04860, phage portal protein
21	Complement 2282578-2284233	551	PHAGE_Clostr_phiCD6356_NC_015262: putative terminase large subunit (2.78e-150)	COG4626, phage terminase-like protein; pfam03354, phage terminase
22	Complement 2284214-2284768	184	PHAGE_Clostr_phiCD6356_NC_015262: putative terminase small subunit (4.73e-44)	
23	Complement 2284871-2285197	108	PHAGE_Lister_LP_030_2_NC_021539: HNH homing endonuclease (1.47e-19)	Cd00085, HNH nucleases; smart00507, HNH nucleases; pfam01844, HNH endonuclease
24	Complement 2285185-2285502	105	PHAGE_Clostr_phiCT19406A_NC_030950: hypothetical protein (7.52e-10)	
25	Complement 2285656-2286198	180	PHAGE_Clostr_phiCD6356_NC_015262: putative site-specific recombinase (6.75e-63)	Cd01192, uncharacterized site-specific tyrosine recombinase; cd01185, integrase IntN1 of Bacteroides mobilizable transposon NBU1 and similar proteins; cd00397, DNA breaking-rejoining enzymes (1.67e-16); pfam00589, phage integrase family
26	Complement 2286195-2286431	78	conserved domain protein (0.0)	
27	Complement 2286475-2286615	46	hypothetical protein (0.0)	
28	Complement 2286609-2286998	129	PHAGE_Clostr_phiCT453A_NC_028991: hypothetical protein (2.01e-27)	TIGR01637, phage transcriptional regulator
29	Complement 2287923-2288141	72	hypothetical protein (0.0)	
30	Complement 2288167-2288352	61	PHAGE_Cellul_phi18:3_NC_021794: hypothetical protein (5.30e-06)	
31	Complement 2288372-2288584	70	PHAGE_Clostr_phiCTC2B_NC_030951: hypothetical protein (8.01e-26)	

32	Complement 2288715-2288981	88	hypothetical protein (0.0)	
33	Complement 2289006-2289161	51	hypothetical protein (0.0)	
34	Complement 2289167-2289280	37	hypothetical protein (0.0)	
35	Complement 2289342-2290208	288	PHAGE_Clostr_vB_CpeS_CP51_NC_021325: hypothetical protein (6.49e-40)	Pfam13730, helix-turn-helix domain
36	Complement 2290324-2291475	383	PHAGE_Staphy_EW_NC_007056: ORF013 (1.26e-11)	Cd16396, nucleoid occlusion protein; TIGR04285, nucleoid occlusion protein; cd16407, ParB N-terminal, parA-binding, -like domain of bacterial and plasmid parABS partitioning systems
37	Complement 2291460-2292215	251	PHAGE_Natria_PhiCh1_NC_004084: putative plasmid partitioning protein Soj (1.34e-22)	COG1192, cellulose biosynthesis protein BcsQ; pfam13614, AAA domain; cd02042, partition proteins ParAB family
38	Complement 2292306-2292449	47	hypothetical protein (0.0)	
39	Complement 2292437-2292685	82	PHAGE_Paenib_Fern_NC_028851: helix-turn-helix domain transcriptional regulator (1.82e-10)	Pfam12728, helix-turn-helix domain
40	Complement 2292698-2293129	143	putative prophage LambdaCh01, transcriptional regulator (0.0)	Pfam13560, Helix-turn-helix domain; smart00530, Helix-turn-helix XRE-family like proteins; cd00093, Helix-turn-helix XRE-family like proteins
41	Complement 2293099-2293212	37	hypothetical protein (0.0)	
42	Complement 2293266-2293460	64	PHAGE_Bacill_vB_BhaS_171_NC_030904: hypothetical protein (4.03e-05)	Smart00530, helix-turn-helix XRE-family like proteins; pfam01381, helix-turn-helix ; cd00093, helix-turn-helix XRE-family like proteins
43	2293596-2293940	114	PHAGE_Lactob_iLp84_NC_028783: repressor (1.76e-13)	Smart00530, helix-turn-helix XRE-family like proteins; cd00093, helix-turn-helix XRE-family like proteins; pfam01381, helix-turn-helix
44	Complement 2293966-2295045	359	PHAGE_Clostr_c_st_NC_007581: metallo beta-lactamase family protein (3.92e-103)	COG2333, metal-dependent hydrolase; cd07731, competence protein ComA, ComEC and related proteins; TIGR00361, DNA internalization-related competence protein ComEC/Rec2
45	Complement 2295201-2295689	162	PHAGE_Strept_phiARI0923_NC_030946: hypothetical protein (2.65e-07)	COG5263, glucan-binding domain; pfam0147, putative cell wall binding repeat
46	2296026-2297078	350	PHAGE_Lactob_Lj965_NC_005355: hypothetical protein (1.77e-81)	
47	2297154-2297486	110	ProFAR isomerase associated superfamily (0.0)	Cd06555, ASC-1 homology domain, subfamily similar to *Pyrococcus furiosus* Pf0470; COG4043, ASC-1 homology (ASCH) domain, predicted RNA-binding domain; smart01022, the ASCH domain adopts a beta-barrel fold similar to that of the PUA domain
48	Complement 2297513-2298052	179	conserved hypothetical protein (0.0)	Pfam13207, AA domain (8.70e-20); COG2019, archaeal adenylate kinase; cd01428, adenylate kinase
49	Complement 2298063-2298440	152	PHAGE_Lactob_Lj965_NC_005355: hypothetical protein (3.17e-39)	COG4933, predicted transcriptional regulator, contains an HTH and PUA-like domains; PRK12279, 50S ribosomal protein L22/unknown domain fusion protein; smart01022, the ASCH domain adopts a beta-barrel fold similar to that of the PUA domain
	2298649-2299146	165	PHAGE_Clostr_phiCT453A_NC_028991: hypothetical protein (4.58e-13)	Pfam06114, IrrE N-terminal-like domain

50	2299251-2300399	382	PHAGE_Clostr_phiMMP01_NC_028883: putative integrase (2.71e-124)	Cd01189, C-terminal catalytic domain of integrases from bacterial phages and conjugate transposons; cd00397, DNA breaking-rejoining enzymes, C-terminal catalytic domain; pfam00589, phage integrase family
51	Complement 2300481-2301359	292	PHAGE_Gordon_Utz_NC_030921: hypothetical protein (7.87e-17)	TIGR02225, tyrosine recombinase XerD, the phage integrase family; COG4974, site-specific recombinase XerD; PRK00283, site-specific tyrosine recombinase XerD
	Complement 2293266-2293460	110	*Clostridium botulinum*, stage II sporulation protein M (2e-143)	TIGR02831, stage II sporulation protein M; pfam01944, stage II sporulation protein M; COG1300, uncharacterized membrane protein SpoIIM

Table S3. Summary of lytic activity of LysB endolysin against representative bacterial strains.

No.	Species	Lysis
1.	*Clostridium sporogenes* ATCC 7955	+
2.	*Clostridium intestinale* ATCC 49213	+
3.	*Clostridium perfringens* Cp39	+
4.	*Clostridium perfringens* JGS1504	+/−
5.	*Bacillus cereus* ATCC 13061	+
6.	*Bacillus megaterium* ATCC 14581	+
7.	*Bacillus mycoides* KPD 15	+
8.	*Bacillus thuringiensis* KPD 114	+
9.	*Staphylococcus aureus* ATCC 25923	+
10.	*Deinococcus radiodurans* ATCC 13939	+
11.	*Bacillus pumilus* KPD 181	−
12.	*Bacillus subtilis* ATCC 6633	−
13.	*Escherichia coli* MG1655	−
14.	*Listeria monocytogenes* KPD 1326	−
15.	*Micrococcus luteus* ATCC 7468	−
16.	*Streptococcus pyogenes* KPD 457	−
17.	*Salmonella enterica* serovar Panama KPD 101	−
18.	*Thermus flavus* MAT 1087	−
