# Supplementary figures and images for: Novel Lytic Enzyme of Prophage Origin from Clostridium botulinum E3 Strain Alaska E43 with Bactericidal Activity against Clostridial Cells
Source: Int J Mol Sci. 2021 Sep 2;22(17):9536. doi: 10.3390/ijms22179536 (PMC8430805; doi:10.3390/ijms22179536)

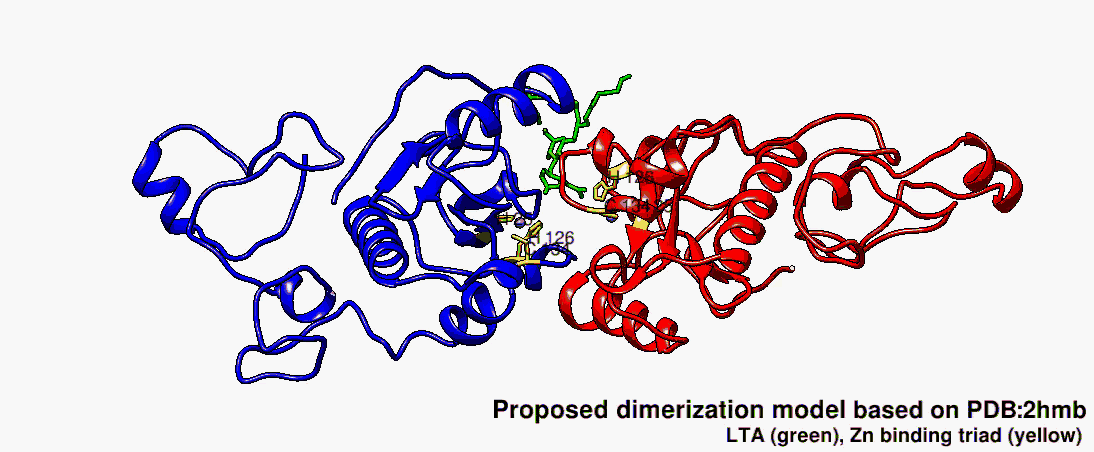

Supplement: Supplementary file 1 [file ijms-22-09536-s001.zip › Supplementary movie 1.gif]
